# Maintaining Self-Control in Chaos: The Protective Role of Sense of Power

**DOI:** 10.5334/pb.1497

**Published:** 2026-09-18

**Authors:** Yifei Feng, Xinyuan Guo, Xinya Huang, Sujie Meng, Ning Liu

**Affiliations:** 1 Faculty of Psychology, Shandong Normal University, Jinan, China; 2 Shandong Provincial Key Laboratory of Brain Science and Mental Health, Jinan, China; 3 School of Psychology, Central China Normal University, Wuhan, China

**Keywords:** self-control, disordered environment, sense of power, power

## Abstract

As a crucial mechanism for regulating human behavior, self-control is influenced by both internal individual factors and external environmental factors. Existing research has established that disordered environments can impair an individual’s self-control, but exploration of moderating factors remains insufficient. This paper proposes that the sense of power is a significant moderating factor in the relationship between disordered environments and self-control. Through experiments employing an intertemporal choice task, a breath-holding task, and a Stroop task, this study verified both the impairing effect of disordered environments on self-control and the moderating role of the sense of power. The results demonstrate that disordered environments harm the willingness to exert self-control, behavioral performance, and inhibitory control. The self-control of individuals with a high trait sense of power was not negatively affected by disordered environments. Furthermore, elevating the state sense of power can protect self-control from the detrimental effects of disordered environments. These findings provide a new perspective for understanding the boundary conditions of, and interventions for, the impairing effect of disordered environments on self-control.

## Introduction

Self-control is defined as the process by which individuals actively override impulses and pursue long-term goals when faced with conflicts between immediate temptations and distant, valued outcomes (Duckworth, Gendler, & Gross, 2016; Hennecke & Bürgler, 2023). This definition reflects the emphasis in self-control research on individuals’ internal processes. However, self-control is not solely determined by internal, dispositional factors (Duckworth et al., 2016; Kotabe & Hofmann, 2015). Environmental factors, particularly disordered physical environments, also play a meaningful role.

A growing body of research indicates that disordered environments impair individual state self-control. With respect to volitional choice, disordered environments increase individuals’ preference for smaller immediate rewards (Fan et al., 2012) and promote the selection of unhealthy foods (Meersseman, Geuens, & Vermeir, 2023). With respect to behavioral performance, they increase impulsive purchasing (Chae & Zhu, 2014) and norm-violating behaviors such as littering and rule-breaking (Keizer, Lindenberg, & Steg, 2008). The most prominent theoretical accounts draw on the resource depletion model of self-control and the sense of control theory. These accounts posit that disordered environments deplete self-control resources and undermine individuals’ sense of control, thereby impairing subsequent self-control behavior (Baumeister et al., 1998; Baumeister, Vohs, & Tice, 2007; Chae & Zhu, 2014; Heatherton & Wagner, 2011; Kotabe, 2014). Furthermore, researchers have found that the detrimental effects of disordered environments extend to the cognitive level, manifesting as impairments in attention and executive functions (Niedernhuber, Kastenmueller, & Fischer, 2014; Zheng, 2016). However, this body of research has focused primarily on the negative consequences of disordered environments while neglecting moderating factors. It remains unclear what individual differences and intervention strategies might shape this effect, which limits a deeper understanding of the phenomenon and impedes the development of effective prevention and intervention efforts.

To address these gaps, the present research examines the moderating role of sense of power to identify the boundary conditions and potential intervention pathways for the detrimental effect of disordered environments on self-control. Sense of power refers to an individual’s perceived capacity to control themselves, others, and outcomes (Galinsky, Gruenfeld, & Magee, 2003). It can be distinguished into state sense of power, which is situationally determined, and trait sense of power, which reflects a stable individual disposition (Anderson et al., 2012; Galinsky et al., 2003). We propose that sense of power moderates the detrimental effect of disordered environments on self-control. Specifically, individuals high in trait power will be immune to the detrimental effects of disordered environments on self-control, and boosting state sense of power will protect self-control from the harmful effects of disordered environments. Two main lines of reasoning support these predictions.

First, research demonstrates a complex bidirectional relationship between power and self-control. Power promotes self-control (Garbinsky, Klesse, & Aaker, 2014; Joshi & Fast, 2013), and self-control in turn facilitates the acquisition and display of power (McIntyre, von Hippel, & Barlow, 2016; Wu, Smallman, & Smith, 2024).

Second, three theoretical perspectives converge to suggest a moderating role for sense of power: self-control resources, sense of control, and executive functions. With respect to self-control resources, according to the situated focus theory of power, high-power individuals possess greater cognitive flexibility and are better able to allocate limited attentional resources to important tasks (Guinote, 2017). This suggests that high-power individuals may respond more flexibly to self-control demands when self-control resources are constrained in a disordered environment. With respect to sense of control, sense of power is grounded in sense of control, and high power is generally associated with a heightened sense of control (Guinote, 2007). When disordered environments undermine individuals’ sense of control (Kotabe et al., 2016), individuals high in trait power may be better equipped to resist this effect. Boosting state sense of power may similarly attenuate it. With respect to executive functions, self-control is closely linked to executive functions (Kotabe & Hofmann, 2015). Research has shown that disordered environments impair executive functions, including attentional dispersion (Niedernhuber, Kastenmueller, & Fischer, 2014) and weakened inhibitory control (Zheng, 2016). DeWall et al. (2011) found that boosting state sense of power improves self-control performance through its effects on executive functions. This suggests that sense of power may help individuals resist the negative impact of disordered environments on executive functions and thereby maintain self-control.

Taken together, whether considered from the perspective of resource depletion and sense of control, or from an executive function perspective, sense of power can moderate the impact of disorder on self-control.

Based on this reasoning, the present research proposes that **disordered environments impair self-control (H1)**, and that **sense of power moderates this detrimental effect (H2)**. Specifically, **individuals high in trait power will not be adversely affected by disordered environments in their self-control (H2a), and boosting state sense of power will attenuate the detrimental effect of disorder on self-control (H2b)**.

## Research Overview

The present research tests the above hypotheses through three progressively deepening studies, employing an intertemporal choice task, a breath-holding task, and a Stroop task, respectively.

Study 1 aims to provide an initial examination of whether disordered environments impair self-control, thereby laying the groundwork for Study 2 and Study 3, which investigate the moderating role of sense of power. Monitoring and resolving conflicts between temptations and valued long-term goals is central to successful self-control and is broadly implicated across self-control processes (Duckworth, Milkman, & Laibson, 2018; Hennecke & Bürgler, 2023; Kotabe & Hofmann, 2015). Study 1 therefore uses an intertemporal choice task to capture individuals’ willingness to override short-term impulses in favor of long-term goals (Berns et al., 2007; Fujita et al., 2006; Guan & He, 2018).

Conflict monitoring and the intention to exert control are integral components of the self-control process (Magen & Gross, 2010; Kotabe & Hofmann, 2015). What matters more, however, is whether individuals can translate this intention into actual behavior that overcomes immediate impulses. Study 2 addresses this limitation by employing a breath-holding task, which requires participants to suppress the urge to breathe in order to prolong breath-holding duration. Like the cold pressor task, taste test, and handgrip task, breath-holding is a behavioral measure of persistence (Dreves et al., 2020; Fujita et al., 2006; Mitchell, 2004). This task captures a more ecologically representative form of self-control and is used to examine whether individuals high in trait power are immune to the detrimental effects of disordered environments on self-control (H2a), thereby identifying a boundary condition of this effect.

Finally, compared with examining individual differences, identifying effective pathways to counteract the negative impact of disordered environments holds greater practical significance, and Study 3 explores this question more deeply. Both the volitional aspect captured by intertemporal choice and the behavioral persistence reflected in actual performance share a common cognitive foundation: the inhibition of prepotent responses, which is subserved by executive functions (Diamond, 2013; Hofmann, Schmeichel, & Baddeley, 2012; Van Hove et al., 2020). Inhibitory control is a core component of executive functions and reflects self-control at the cognitive level (Diamond, 2013). Study 3 therefore employs the Stroop task (Chae & Zhu, 2014) to test the interventional effect of boosting state sense of power (H2b).

Data entry was performed using SPSS 25. Statistical analyses were conducted using SPSS 25 and the statsmodels package in Python 3.13. All study materials, datasets, and analysis code are available on the Open Science Framework (OSF) via this anonymous link: https://osf.io/dq7k9/?view_only=3ec3a52d8a47493c936974be23cb7dc5.

## Study 1

Study 1 employed an intertemporal choice task to preliminarily examine the impact of disordered environments on self-control, providing a foundation for subsequent research. Intertemporal choice reflects an individual’s pursuit of and persistence toward long-term goals and has been widely used as a behavioral measure of self-control (De Ridder et al., 2012; Fujita et al., 2006; Macaskill et al., 2019).

### Participants and Research Design

The sample size was determined a priori using G*Power 3.1 (Faul et al., 2007). Previous studies have typically found a small to moderate effect size for the impact of environmental disorder on self-control (Chae & Zhu, 2014; Meersseman et al., 2023; Vohs et al., 2013). Therefore, we conducted an a priori power analysis using an estimated effect size of *d* = 0.50, with α = .05 and power = .80. The analysis indicated a minimum required sample size of 102 participants. A total of 135 university students participated in the experiment. After excluding data from 5 participants for whom the environmental order manipulation was unsuccessful, the final valid sample consisted of 130 participants (107 female), with a mean age of 20.42 ± 1.48 years. Participants were randomly assigned to either the orderly group (*n* = 65) or the disordered group (*n* = 65).

This study employed a single-factor, between-subjects design. The independent variable was environmental order (disordered vs. orderly), manipulated using scene pictures. The dependent variable was the minimum acceptable delayed amount in the intertemporal choice task, where a higher amount indicates poorer self-control (Paglieri et al., 2013).

### Experimental Procedure and Materials

**Figure 1:**
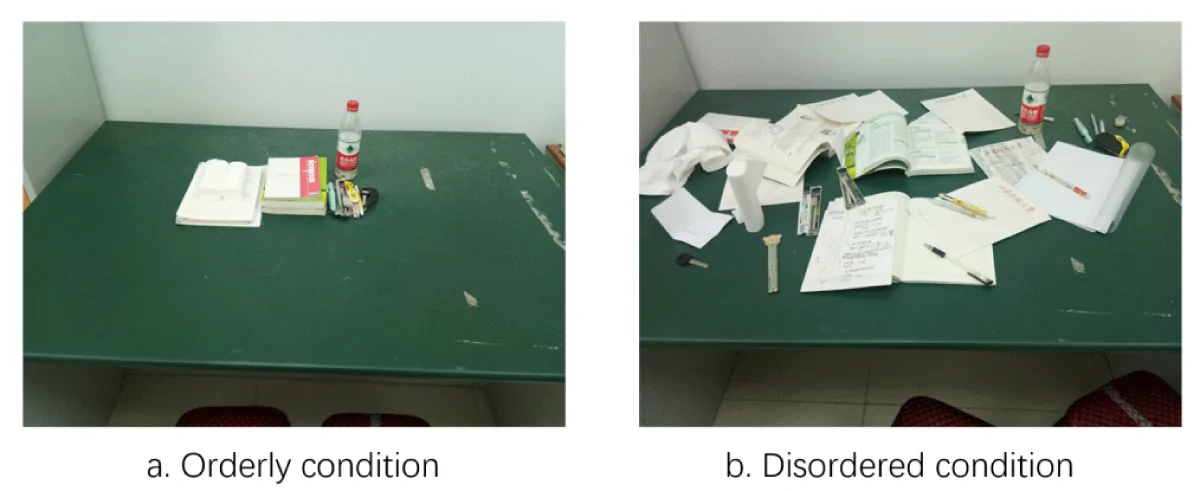
Experimental Materials for Study 1.

The experimental procedure commenced with the presentation of scene pictures to manipulate environmental order. The picture shows the laboratory desktop under two conditions: (a) orderly and (b) disordered (see Figure 1). Participants were instructed to imagine themselves within the depicted scenario and to provide a 3-minute written description of the scene.

Following this, participants completed the intertemporal choice task. The task was adapted from Van den Bergh et al. (2008). They were informed that as compensation for participating in a campus documentary filming session, they could choose to receive 50 CNY either immediately or a higher amount after a one-week delay. The key dependent variable was their response to the question: “If you could only receive the payment after one week, what is the minimum amount you would find acceptable?” The higher the amount participants reported, they were more strongly drawn to immediate rewards thus exhibited poorer self-control (Ainslie & Haslam, 1992; Frederick et al., 2002; Fujita et al., 2006; Thaler, 1981).

Subsequently, participants completed a manipulation check for the environmental order manipulation. This check consisted of two items (α = 0.96), rated on a 7-point scale, with higher scores indicating a greater perception of disorder in the scene pictures (Chae & Zhu, 2014).

Following the manipulation check, several control variables were measured. These covariates were selected on theoretical grounds. (1) Emotional state was assessed using the Positive and Negative Affect Schedule (PANAS; α = .85 for positive affect, α = .84 for negative affect; 10 items each on a 5-point scale). Prior research has shown that affect can influence self-control performance (Fishbach & Labroo, 2007), and the experimental manipulation of environmental disorder may incidentally evoke affective responses. (2) Trait self-control was measured using the 13-item Self-Control Scale (SCS; α = .73; 5-point scale), as individuals’ baseline capacity for self-regulation may confound the effect of environmental disorder (De Ridder et al., 2012; Tangney et al., 2004). (3) Trait orderliness was assessed using a two-item habits questionnaire (α = .88; 7-point scale) designed to capture individuals’ inherent preference for neatness, because participants high in trait orderliness may be differentially sensitive to environmental disorder (Li et al., 2020). For all three measures, higher scores indicate higher levels of the respective construct.

Finally, demographic information was collected from the participants. The experimenter then provided compensation, thanked the participants, and concluded the session.

### Results and Analysis

#### Common Method Bias and Manipulation Check

Harman’s single-factor test was conducted on the questionnaire data to assess common method bias. The first factor accounted for 17.64% of the variance (below the 40% threshold) prior to rotation, indicating that common method bias was not a serious concern in this study.

An independent-samples *t*-test was performed with the environmental order condition as the independent variable and the rating of the scene’s orderliness as the dependent variable. The results revealed that the disorder rating was significantly higher in the disordered environment group (*M* = 5.75, *SD* = 1.06) than in the orderly environment group (*M* = 1.98, *SD* = 0.99), *t*(128) = 20.88, *p* < .001, Cohen’s *d* = 3.66. This confirms that the manipulation of environmental order was successful.

#### The Effect of Disordered Environment on Self-Control

An independent-samples *t*-test was first conducted to examine the effect of environmental order on self-control without covariate adjustment. The dependent variable was the minimum acceptable delayed amount. The results revealed that the minimum acceptable delayed amount was significantly higher in the disordered environment group (*M* = 80.18, *SD* = 24.65) than in the orderly environment group (*M* = 72.55, *SD* = 15.34), *t*(128) = 2.119, *p* = .036, Cohen’s *d* = 0.372.

To assess the robustness of this effect, a one-way analysis of covariance (ANCOVA) was subsequently conducted, including positive affect, negative affect, trait self-control, and trait orderliness as covariates. All four covariates were balanced across the two environmental conditions (*p*s > .22), confirming successful randomization. The main effect of environmental order remained significant, *F*(1, 124) = 5.17, *p* = .025, *η_p_*^2^ = 0.04. None of the covariates were statistically significant: positive affect, *F*(1, 124) = 1.36, *p* = .25, *η_p_*^2^ = 0.01; negative affect, *F*(1, 124) = 1.47, *p* = .23, *η_p_*^2^ = 0.01; trait self-control, *F*(1, 124) = 1.38, *p* = .24, *η_p_*^2^ = 0.01; trait orderliness, *F*(1, 124) = 2.35, *p* = .13, *η_p_*^2^ = 0.02.

**Figure 2:**
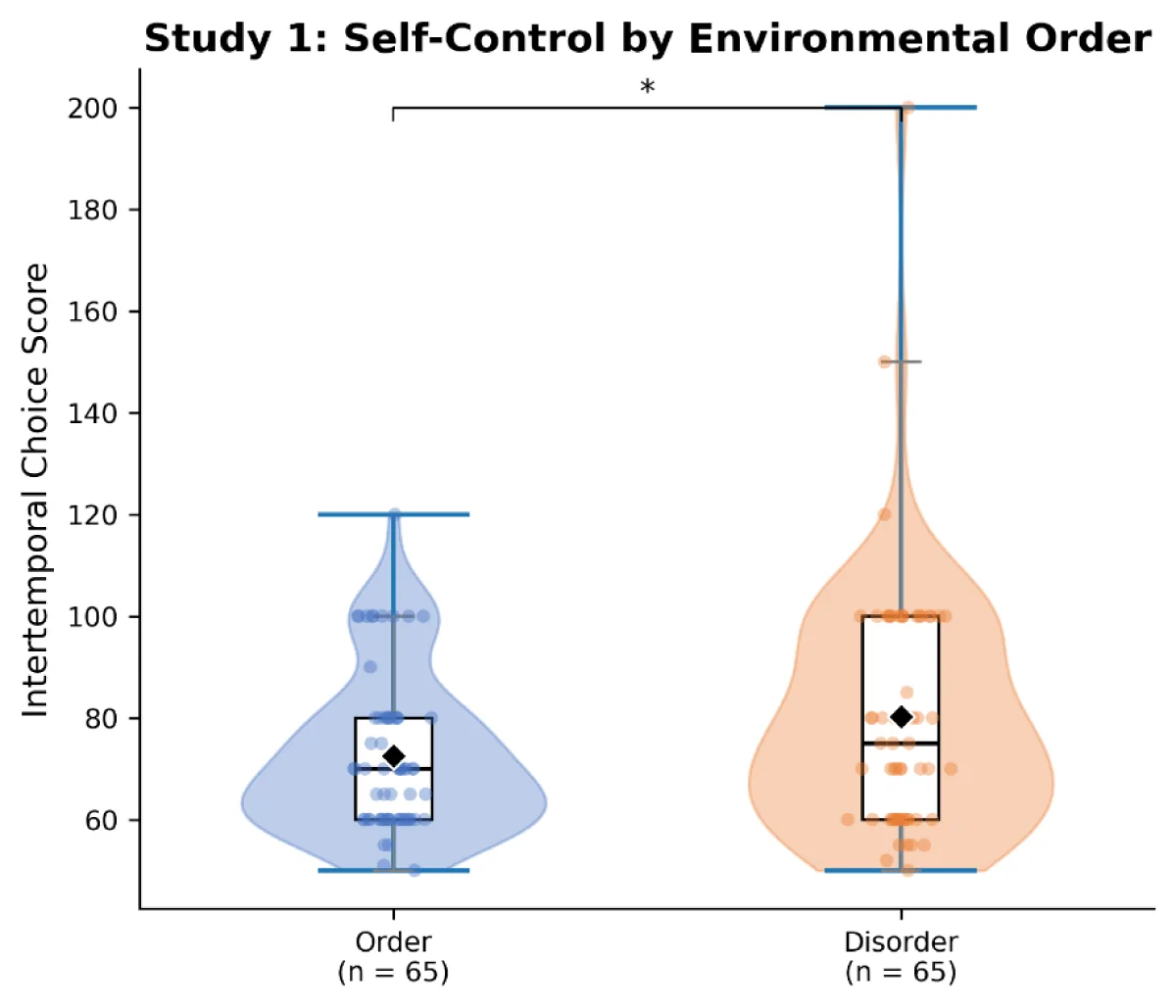
Effect of Environmental Order on Intertemporal Choice.

The results of Study 1 indicate that individuals in a disordered environment exhibited a weaker willingness to exert self-control, providing preliminary support for Hypothesis 1 (see Figure 2). However, possessing the intention to exercise self-control represents only one component of the self-control process; translating this intention into actual behavior is ultimately what makes self-control effective. Study 2 further examines the manifestation of this detrimental effect on concrete self-control behaviors and investigates its boundary condition.

## Study 2

Study 2 had two primary objectives. First, it aimed to replicate the effect of a disordered environment on self-control at the behavioral level by utilizing a breath-holding task. Breath-holding is a persistence task (Vohs & Schmeichel, 2003). Persistence tasks require overcoming physical discomfort and have been widely used as a behavioral measure of self-control (Mann & Ward, 2025). Second, it sought to further investigate the boundary conditions of this influence. In contrast to Study 1, which used pictorial stimuli to induce a sense of disorder, participants in Study 2 were placed in an actual disordered physical environment.

### Participants and Research Design

The sample size was determined a priori using G*Power 3.1 (Faul et al., 2007). Consistent with prior research (Chae & Zhu, 2014; Joshi & Fast, 2013; Smith et al., 2008), we assumed a medium effect size (*η*² = 0.10). With α = .05, a sample of 119 participants was calculated to be necessary to achieve 95% statistical power (1–*β*). A total of 172 university students were initially recruited and completed the trait sense of power prescreening. Of these, 5 participants did not complete the full screening questionnaire and 8 participants did not complete the experiment, yielding 159 participants who proceeded to the main experiment. After further excluding data from 10 participants whose breath-holding times exceeded ±3 standard deviations, the final valid sample consisted of 149 participants (33 males, 116 females), with a mean age of 19.79 ± 1.09 years.

This study employed a 2 (environmental order) between-subjects design, with trait sense of power included as a continuous, mean-centered moderator. Breath-holding duration varies considerably due to individual differences in lung capacity, which is influenced by factors such as gender, age, athletic ability, smoking status, and other variables (Hogan et al., 2015). In this study, we therefore used the difference between the two breath-holding durations as the dependent variable, thereby controlling for the influence of individual differences in physiological capacity on the experimental outcomes (Li et al., 2020; Muraven et al., 1998; Vohs & Schmeichel, 2003).

**Figure 3:**
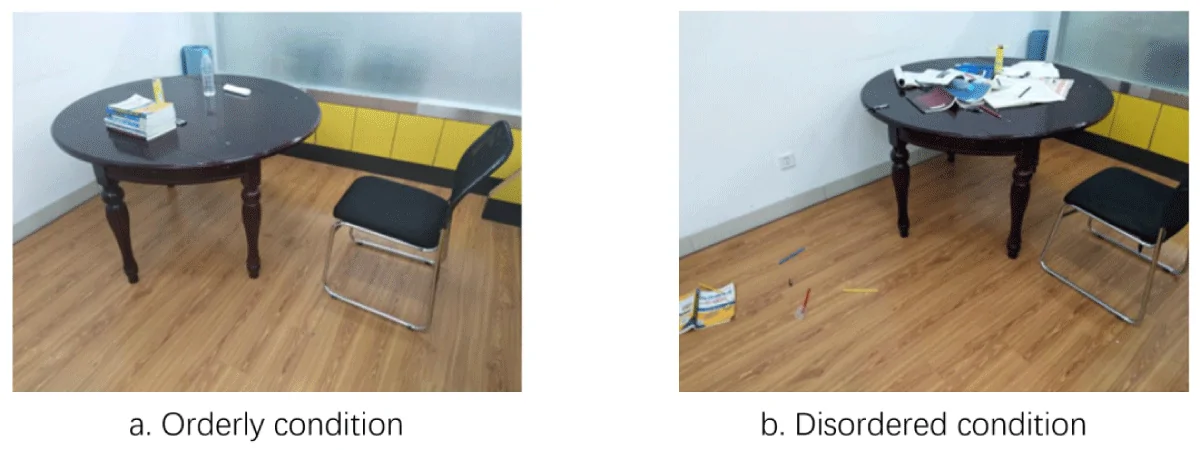
Experimental Conditions in Study 2.

Trait sense of power was measured using the General Sense of Power Scale (Anderson, John, & Keltner, 2012), which consists of 8 items (α = 0.64) rated on a 7-point scale, with higher scores indicating a stronger trait sense of power. Participants were then randomly assigned to either the disordered or orderly environment condition (see Figure 3). In the orderly condition, the items were neatly arranged on the desk (a), whereas in the disordered condition, they were scattered messily on both the desk and the floor (b).

### Experimental Procedure and Materials

Upon being randomly assigned to either the orderly or disordered experimental environment, participants were informed that the researchers were collecting data on the average respiratory capacity of university students. They were instructed to complete two breath-holding trials, meaning they should hold their breath until they genuinely could no longer continue. The experimenter recorded the breath-holding time for each trial, and the average of the two trials was taken as the T1 measure.

Subsequently, under the pretext of needing to prepare experimental materials, the experimenter left the participant alone in the disordered or orderly environment for 1 minute and 30 seconds. This allowed the participant to notice and be exposed to the experimental setting. After the experimenter returned, the participant completed the breath-holding task twice more. The average of these two trials was taken as the T2 measure.

Following this, participants completed a manipulation check for the environmental order. This check consisted of two items (α = 0.91; e.g., “How disordered do you think the current scene is?”), rated on a 7-point scale, with higher scores indicating a greater perception of disorder in the experimental environment (Chae & Zhu, 2014).

Next, measures for control variables were administered, consistent with Study 1 and the unified covariate strategy. These included: Emotional state, measured using the Positive and Negative Affect Schedule (Positive Affect α = 0.83, Negative Affect α = 0.90). Trait self-control (α = 0.84). Trait orderliness (α = 0.80).

Finally, demographic information was collected. The experimenter then provided compensation, thanked the participant, and concluded the session.

### Results and Analysis

#### Common Method Bias and Manipulation Check

Harman’s single-factor test was conducted on the questionnaire data. The first factor accounted for 27.71% of the variance (below the 40% threshold) prior to rotation, indicating that common method bias was not a serious concern in this study.

An independent-samples *t*-test was performed with the environmental order condition as the independent variable and the rating of the experimental environment’s orderliness as the dependent variable. The results demonstrated that the disorder rating was significantly higher in the disordered environment group (*M* = 5.76, *SD* = 1.00) than in the orderly environment group (*M* = 1.95, *SD* = 0.84), *t*(147) = 25.23, *p* < .001, Cohen’s *d* = 4.13. This confirms that the manipulation of environmental order was successful.

#### The Effect of Environmental Order on Self-Control

We first conducted an analysis of variance (ANOVA) to examine the unadjusted effect of environmental order on the difference between T2 and T1 breath-holding durations. Subsequently, an ANCOVA was conducted including positive affect, negative affect, trait self-control, and trait orderliness as covariates.

An analysis of variance (ANOVA) was conducted with environmental order as the independent variable and the difference in breath-holding time (T2-test minus T1-test) as the dependent variable. The results revealed that the increase in breath-holding time was significantly smaller in the disordered environment (*M* = 4.02, *SD* = 9.05) than in the orderly environment (*M* = 7.08, *SD* = 8.69), *F*(1, 147) = 4.43, *p* = .037, *η_p_*^2^ = 0.03.

Scores for positive affect, negative affect, trait self-control, and trait orderliness were then included as covariates in an ANCOVA. All four covariates were balanced across the two environmental conditions (*p*s > .07). The results indicated that the main effects of all covariates were non-significant: trait orderliness, *F*(1, 143) = 0.48, *p* = .49, *η_p_*^2^ < 0.01; trait self-control, *F*(1, 143) = 3.10, *p* = .08, *η_p_*^2^ = 0.02; positive affect, *F*(1, 143) = 0.63, *p* = .43, *η_p_*^2^ < 0.01; negative affect, *F*(1, 143) = 0.57, *p* = .45, *η_p_*^2^ < 0.01. After controlling for these covariates, the main effect of environmental order remained significant, *F*(1, 143) = 5.57, *p* = .02, *η_p_*^2^ = 0.04. These results demonstrate the detrimental effect of a disordered environment on self-control behavior, thereby providing further support for Hypothesis H1.

#### The Moderating Role of Sense of Power

A moderation analysis was conducted using multiple regression with environmental order (coded as 0 = orderly, 1 = disordered), trait sense of power (mean-centered, continuous), and their interaction term as predictors, and the difference in breath-holding time as the dependent variable. Simple slope analyses were performed at +1 *SD* and –1 *SD* of the continuous power moderator (Aiken & West, 1991). To verify the robustness of the moderation effect, an ANCOVA was also conducted with the same four covariates (positive affect, negative affect, trait self-control, trait orderliness).

The unadjusted model (without covariates) revealed a significant main effect of environmental order, *β* = –3.31, *p* = .025. The main effect of trait sense of power was not significant, *β* = 0.91, *p* = .454. Critically, the Environment × Trait Sense of Power interaction was not significant, *β* = 1.13, *p* = .559.

To assess the robustness of these findings, the same regression model was re-estimated with positive affect, negative affect, trait self-control, and trait orderliness included as additional covariates. In this adjusted model, trait self-control emerged as a significant covariate (*β* = –3.29, *p* = .027), indicating its potential involvement in how environmental order and sense of personal power influence self-control. The other three covariates were not significant (positive affect, *β* = 0.01, *p* = .952; negative affect, *β* = 0.15, *p* = .226; trait orderliness, *β* = –0.29, *p* = .607). The Environment × Trait Sense of Power interaction remained non-significant, *β* = 0.12, *p* = .950.

Although the overall interaction was not significant, a simple slope analysis was conducted to probe the pattern of effects at different levels of trait sense of power. At low levels of trait sense of power (–1 *SD*), the effect of environmental disorder on breath-holding time approached significance, *β* = –4.18, *p* = .050. At high levels of trait sense of power (+1 *SD*), the effect was not significant, *β* = –2.43, *p* = .242. This pattern is directionally consistent with the hypothesis that individuals with higher trait sense of power are less susceptible to the detrimental effect of environmental disorder on self-control. However, because the overall interaction was not significant (*p* = .559), these simple slope results should be interpreted with caution.

**Figure 4:**
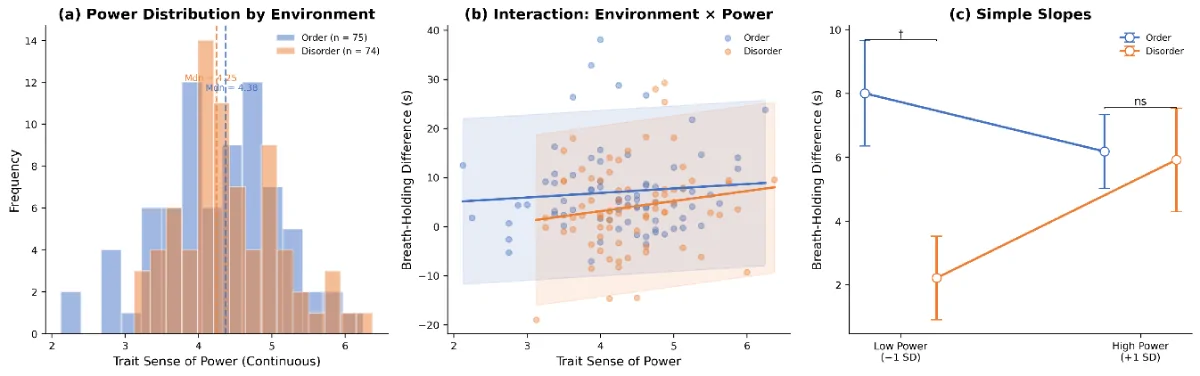
The Moderating Role of Trait Sense of Power.

Building on Study 1, Study 2 examined a specific behavioral manifestation of self-control—breath-holding endurance. The results provided further support for Hypothesis H1: individuals exposed to a disordered environment exhibited poorer self-control performance than those in an orderly environment. With regard to Hypothesis H2a, the Environment × Trait Sense of Power interaction did not reach significance; however, the pattern of simple slopes was directionally consistent with the prediction that higher trait sense of power buffers against the detrimental effect of environmental disorder (see Figure 4). Finally, given that individuals with high trait sense of power appeared less affected by the disordered environment, a natural question arises: can the experimental elevation of state sense of power similarly attenuate this impairment? Study 3 was designed to address this question.

## Study 3

Study 3 aimed to investigate the moderating role of state sense of power using a Stroop task. The Stroop task captures individuals’ ability to inhibit prepotent responses. It measures inhibitory control, a core component of executive functions, and reflects self-control at the cognitive level (Van Hove et al., 2020).

### Participants and Research Design

The sample size was determined a priori using G*Power 3.1 (Faul et al., 2007). Consistent with prior research (Chae & Zhu, 2014; Joshi & Fast, 2013; Smith et al., 2008), we assumed a medium effect size (*η*² = 0.10). With α = 0.05, a sample of 119 participants was calculated to be necessary to achieve 95% statistical power (1–*β*). A total of 122 university students (107 female) participated in the experiment in exchange for course credit or monetary compensation. Participants ranged in age from 17 to 23 years (*M* = 19.52, *SD* = 1.25) and had normal or corrected-to-normal vision.

Study 3 employed a 2 (Environmental Order: disordered vs. orderly) × 2 (State Sense of Power: low vs. high) between-subjects design. The dependent variables were the interference effect magnitudes for both response time and accuracy in the Stroop task (Diamond, 2013; Rosen et al., 2016; Dvorak, 2024). Specifically, the Response Time Interference Effect Magnitude (RT-IEM) was calculated by subtracting the mean reaction time in the congruent condition from the mean reaction time in the incongruent condition. The Accuracy Interference Effect Magnitude (ACC-IEM) was calculated by subtracting the accuracy rate in the incongruent condition from the accuracy rate in the congruent condition.

Participants were randomly assigned to one of four groups: disordered environment/high state sense of power (*n* = 31), disordered environment/low state sense of power (*n* = 30), orderly environment/high state sense of power (*n* = 31), and orderly environment/low state sense of power (*n* = 30).

### Experimental Procedure and Materials

**Figure 5:**
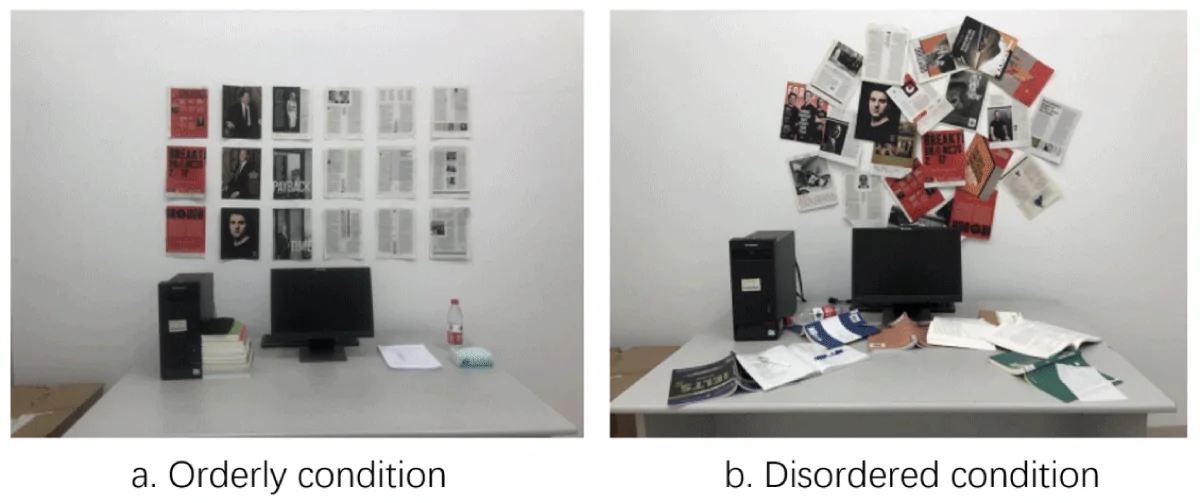
Experimental Conditions in Study 3.

Participants were randomly assigned to either an orderly or disordered experimental environment (see Figure 5). In the orderly condition, items were neatly arranged on the desk and magazine pages were neatly posted on the wall (a). In the disordered condition, these items were scattered messily across the desk and the magazine pages were posted in a disorderly manner on the wall (b).

Under the pretext of needing to prepare experimental materials, the experimenter left the participant alone in the assigned environment for 1 minute and 30 seconds, allowing them to notice the experimental setting.

**Figure 6:**
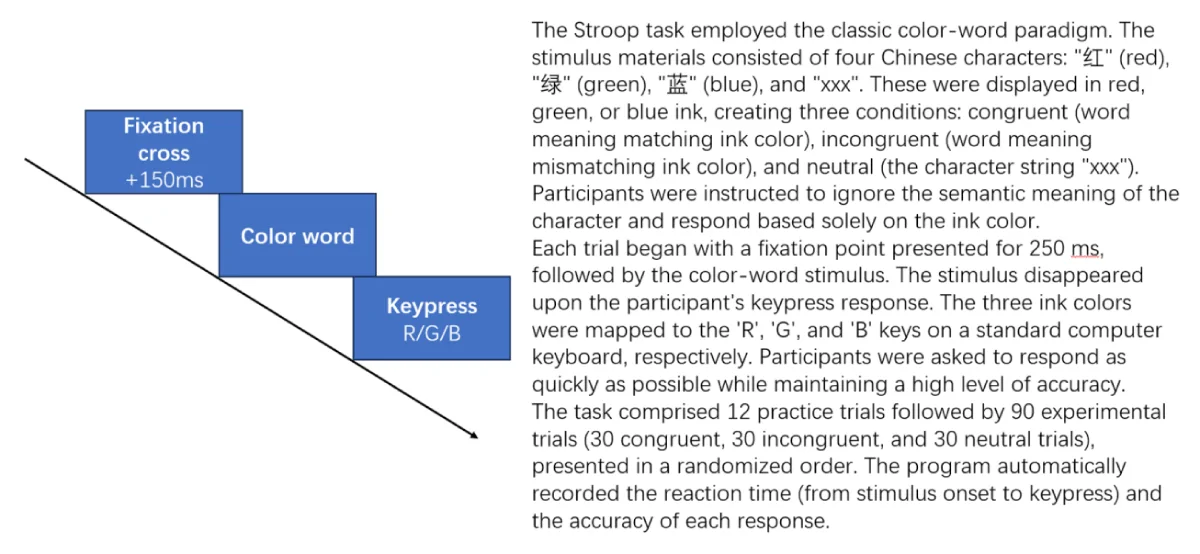
Schematic Diagram of the Stroop Task.

Upon returning, the experimenter conducted a manipulation of state sense of power. Participants in the high state sense of power condition were instructed to recall an experience in which they had power over others, while those in the low state sense of power condition recalled an experience in which someone else had power over them. Following this manipulation, participants completed the Stroop task (see Figure 6).

Subsequently, participants completed a manipulation check for the environmental order, which consisted of two items (α = 0.97), identical to the measure used in Study 2. They also completed a manipulation check for the sense of power, comprising two items (α = 0.76; e.g., “I feel very powerful”), rated on a 7-point scale, with higher scores indicating a stronger perceived sense of power (Kraus et al., 2011).

Following the manipulation checks, and consistent with previous studies, participants completed the following measures: (1) Emotional state was assessed using the Positive and Negative Affect Schedule (PANAS; Positive Affect α = 0.83, Negative Affect α = 0.81). (2) Trait self-control was measured using the Self-Control Scale (SCS; α = 0.79). (3) Trait orderliness was assessed using a two-item measure (α = 0.86, 7-point scale), with higher scores indicating a greater focus on environmental tidiness in daily life. Additionally, participants completed a measure of trait sense of power (Anderson et al., 2012) to control for its potential influence. These five covariates — positive affect, negative affect, trait self-control, trait orderliness, and trait power — were included in supplementary analyses.

Finally, demographic information was collected. The experimenter then provided compensation, thanked the participant, and concluded the session.

### Results and Analysis

#### Manipulation Checks

Descriptive statistics for reaction time and accuracy (shown in Table 1) indicated that participants prioritized accuracy while responding as quickly as possible.

**Table 1:** Descriptive Statistics for Accuracy and Reaction Time in the Stroop Task.


DATA TYPE	ACC		RT (ms)	
	
	INCONGRUENT CONDITION	CONGRUENT CONDITION	CONGRUENT CONDITION	INCONGRUENT CONDITION

Average	0.985	0.948	683.829	796.256

Median	1.000	0.966	656.717	782.408

*SD*	0.024	0.053	128.550	170.166


An independent-samples *t*-test was conducted with the environmental order condition as the independent variable and the disorder rating of the experimental scene as the dependent variable. The results showed that the disorder rating was significantly higher in the disordered environment group (*M* = 11.00, *SD* = 2.27) than in the orderly environment group (*M* = 3.66, *SD* = 2.21), *t*(120) = 18.14, *p* < .001, Cohen’s *d* = 3.31. This confirms the successful manipulation of environmental order.

Another independent-samples *t*-test was conducted with the sense of power condition as the independent variable and the state sense of power manipulation check score as the dependent variable. The results revealed that the score was significantly higher in the high state sense of power group (*M* = 10.03, *SD* = 2.46) than in the low state sense of power group (*M* = 5.73, *SD* = 2.78), *t*(120) = 9.06, *p* < .001, Cohen’s *d* = 1.65. This confirms the successful manipulation of state sense of power.

#### Accuracy Analysis

A two-way analysis of variance (ANOVA) was conducted with environmental order and state sense of power as independent variables and the ACC-IEM as the dependent variable, without covariates (unadjusted). Subsequently, a two-way ANCOVA was conducted including positive affect, negative affect, trait self-control, trait orderliness, and trait power as covariates.

A two-way analysis of variance (ANOVA) was first conducted without covariates, with environmental order and state sense of power as independent variables and the ACC-IEM as the dependent variable. The results revealed a non-significant main effect of state sense of power, *F*(1, 118) = 1.06, *p* = .305, *η_p_*^2^ = 0.009. However, the main effect of environmental order was significant, *F*(1, 118) = 5.26, *p* = .024, *η_p_*^2^ = 0.04. The ACC-IEM was significantly larger in the disordered environment (*M* = 0.05, *SD* = 0.06) than in the orderly environment (*M* = 0.03, *SD* = 0.05). This indicates that the incongruence between word meaning and color caused a greater impairment to accuracy in the disordered environment, thereby supporting Hypothesis H1.

Critically, the interaction between environmental order and state sense of power was significant, *F*(1, 118) = 5.00, *p* = .027, *η*_p_^2^ = 0.04. Simple effects analysis was conducted to probe this interaction. For individuals with low state sense of power, the ACC-IEM was significantly larger in the disordered environment (*M* = 0.07, *SD* = 0.07) compared to the orderly environment (*M* = 0.02, *SD* = 0.05), *F*(1, 118) = 10.26, *p* = .002, *η*_p_^2^ = 0.08. In contrast, for individuals with high state sense of power, there was no significant difference in ACC-IEM between the disordered environment (*M* = 0.03, *SD* = 0.04) and the orderly environment (*M* = 0.03, *SD* = 0.06), *F*(1, 118) = 0.00, *p* = .948. This indicates that by enhancing the state sense of power, an individual’s self-control ability becomes more resistant to the effects of a disordered environment. Therefore, this result supports Hypotheses H2 and H2b.

Next, the robustness of these results was tested by including five covariates (positive affect, negative affect, trait self-control, trait orderliness, and trait power) in an ANCOVA. After controlling for these covariates, the main effect of environment remained significant, *F*(1,113) = 4.57, *p* = 0.035, *η*_p_^2^ = 0.04. The Environment × State Sense of Power interaction became marginally significant for ACC-IEM, *F*(1, 113) = 3.32, *p* = .071, *η*_p_^2^ = 0.03. Among the covariates, trait power was a significant predictor, *F*(1, 113) = 7.10, *p* = .009; negative affect was no longer marginally significant, *F*(1, 113) = 2.35, *p* = .128; the remaining covariates were not significant: positive affect, *F*(1, 113) = 0.25, *p* = .620; trait self-control, *F*(1, 113) = 0.003, *p* = .956; trait orderliness, *F*(1, 113) = 0.29, *p* = .590.

In summary, the accuracy results demonstrate that disordered environments impair individuals’ inhibitory control ability, supporting Hypothesis H1. Furthermore, elevating state sense of power reduced this detrimental effect, supporting Hypotheses H2 and H2b. The attenuated interaction after covariate adjustment suggests that negative affect partially accounts for the observed effect.

#### Reaction Time Analysis

A two-way analysis of variance (ANOVA) was conducted with environmental order and state sense of power as independent variables and the RT-IEM as the dependent variable, without covariates. Subsequently, a two-way ANCOVA was conducted including positive affect, negative affect, trait self-control, trait orderliness, and trait power as covariates.

A two-way analysis of variance (ANOVA) was conducted with environmental order and state sense of power as independent variables and the RT-IEM as the dependent variable. The results showed a non-significant main effect of state sense of power, *F*(1, 118) = 0.03, *p* = .870. The main effect of environmental order was also non-significant: the RT-IEM in the orderly environment (*M* = 107.60 ms, *SD* = 79.29 ms) did not differ significantly from that in the disordered environment (*M* = 117.25 ms, *SD* = 86.76 ms), *F*(1, 118) = 0.42, *p* = .517.

However, the interaction between environmental order and state sense of power was significant, *F*(1, 118) = 5.40, *p* = .022, *η*_p_^2^ = 0.04. Simple effects analysis revealed that for individuals with low state sense of power, the RT-IEM was significantly larger in the disordered environment (*M* = 136.02 ms, *SD* = 94.41 ms) than in the orderly environment (*M* = 91.32 ms, *SD* = 75.91 ms), *F*(1, 118) = 4.46, *p* = .037, *η*_p_^2^ = 0.04. For individuals with high state sense of power, there was no significant difference in RT-IEM between the disordered environment (*M* = 99.10 ms, *SD* = 75.79 ms) and the orderly environment (*M* = 123.36 ms, *SD* = 80.52 ms), *F*(1, 118) = 1.36, *p* = .246.

In the Stroop task, the disordered environment caused a greater increase in reaction time for the incongruent condition among individuals with low state sense of power, again reflecting the impairment of inhibitory control ability by the disordered environment. The lack of a significant difference in RT-IEM across environments for individuals with high state sense of power demonstrates the intervention value of elevating state sense of power, providing further support for Hypotheses H2 and H2b.

After including the same set of five covariates (positive affect, negative affect, trait self-control, trait orderliness, and trait power) in an ANCOVA, the main effect of environment remained non-significant (*p* > .05). The Environment × State Sense of Power interaction remained significant and was slightly strengthened, *F*(1, 113) = 5.69, *p* = .019, *η*_p_^2^ = 0.05. None of the five covariates were significant predictors of RT-IEM: positive affect, *F*(1, 113) = 0.09, *p* = .769; negative affect, *F*(1, 113) = 0.97, *p* = .327; trait self-control, *F*(1, 113) = 0.44, *p* = .509; trait orderliness, *F*(1, 113) = 0.39, *p* = .534; trait power, *F*(1, 113) = 1.07, *p* = .303. The finding that elevating state sense of power can mitigate the detrimental effect of a disordered environment on inhibitory control held after covariate adjustment. Unlike the ACC-IEM results, the RT-IEM interaction was not attenuated by the inclusion of the five covariates, suggesting that this effect is robust.

#### Summary

**Figure 7:**
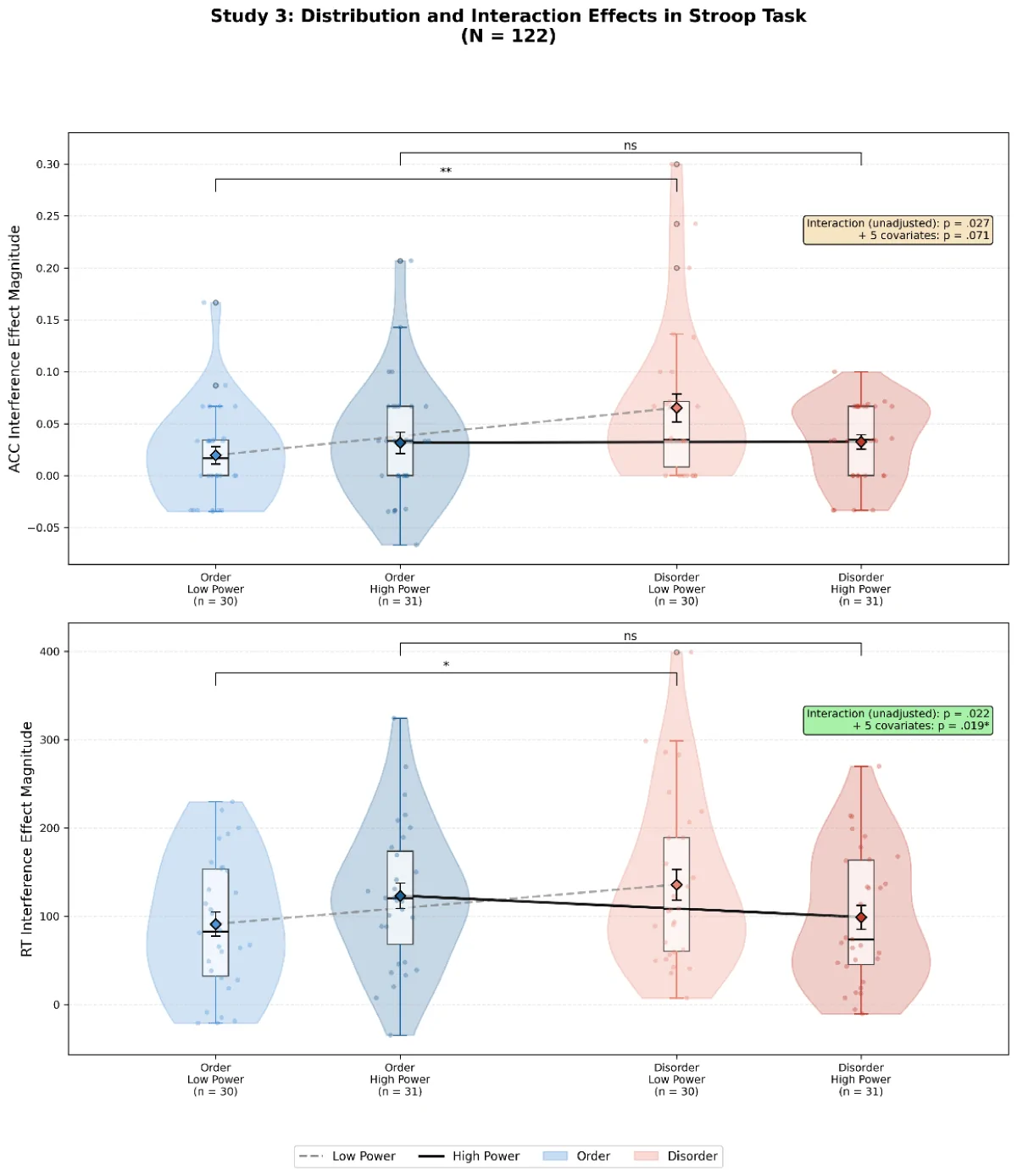
Interaction Between Environmental Order and State Sense of Power on Self-Control.

In summary, using the Stroop paradigm, Study 3 demonstrated that disordered environments impair individuals’ inhibitory control ability. Furthermore, elevating state sense of power mitigated this detrimental effect across both ACC-IEM and RT-IEM. These results support Hypotheses H1, H2, and H2b (see Figure 7).

## General Discussion

The present research investigated the detrimental effect of disordered environments on self-control and examined the moderating role of sense of power. Using an intertemporal choice task, Study 1 demonstrated that disordered environments undermine individuals’ willingness to exert self-control. Study 2, employing a breath-holding task, extended this finding by showing that the detrimental effect goes beyond volitional choice and manifests in concrete self-control behavior, and further identified high trait power as a potential boundary condition of this effect. Finally, Study 3, utilizing a Stroop task, demonstrated that the impairment of self-control by disordered environments also operates at the level of inhibitory control, and that boosting state power can attenuate this impairment.

### Theoretical and Practical Implications

First, the present findings align with prior research in supporting the detrimental effect of disordered environments on self-control (Fan et al., 2012; Chae & Zhu, 2014; Meersseman et al., 2023; Niedernhuber et al., 2014; Zheng, 2016). However, prior findings have been relatively scattered across different levels of analysis. By employing an intertemporal choice task, a breath-holding task, and a Stroop task, the present research demonstrates that disordered environments impair self-control at the volitional, behavioral, and cognitive levels. This yields a more systematic and deeper understanding of the phenomenon. At the volitional level, disordered environments lead individuals to prefer smaller immediate rewards. At the behavioral level, they directly impair overt behavioral performance. At the cognitive level, they undermine inhibitory control, highlighting the critical role of executive functions as a core mechanism of cognitive and behavioral regulation (Diamond, 2013).

Second, while prior research has documented various manifestations of the detrimental effect of disordered environments on self-control (Fan et al., 2012; Chae & Zhu, 2014; Meersseman et al., 2023), it has largely neglected moderating factors. The present research found that sense of power, whether measured as a trait or manipulated as a state, moderates the detrimental effect of disordered environments on self-control. Specifically, high sense of power serves both as a boundary condition that shields individuals from this effect and as a protective factor that can be mobilized through intervention. These findings reveal a moderating factor of this effect for the first time, thereby extending our understanding of the phenomenon.

Furthermore, prior research has offered three largely independent theoretical accounts of this phenomenon: self-control resource depletion, sense of control, and cognitive control (Chae & Zhu, 2014; Kotabe, 2014; Kotabe et al., 2016; Zheng, 2016). The present research reveals an interaction between sense of power and disordered environments. High sense of power simultaneously confers greater cognitive flexibility, a heightened sense of control, and stronger executive functions (DeWall et al., 2011; Guinote, 2007). This suggests that the detrimental effect of disordered environments on self-control cannot be reduced to a single mechanistic pathway. Rather, it likely reflects the synergistic operation of multiple mechanisms. Specifically, disordered environments may impair self-control by draining self-control resources, undermining sense of control, and weakening executive functions. Sense of power buffers against this impairment by strengthening each of these functions. Self-control resources serve as a psychological buffer that absorbs the negative impact of disordered environments. Sense of control shapes individuals’ subjective expectations and experiences. Executive functions play a pivotal role as the underlying cognitive mechanism.

Notably, neuroscience research indicates that executive functions depend on a distributed network of brain regions, including the prefrontal cortex, cingulate cortex, and parietal lobes (Ardila, Bernal, & Rosselli, 2018; Diamond, 2013; Sutin et al., 2022). Boosting sense of power activates prefrontal regions and reduces sensitivity in the anterior cingulate cortex and insula (Guinote, 2007; Schultheiss et al., 2008; Smith et al., 2008). This suggests that the protective effect of sense of power may stem from its ability to sustain the functioning of these brain regions in a disordered environment. However, this physiological mechanism awaits validation through neuroimaging research.

In summary, sense of power protects individuals in disordered environments at the neural and cognitive level through its effects on executive functions, and at the behavioral level through its effects on overt self-control. This highlights the value of power as both a psychological and a social resource for goal-directed behavior.

### Future Research and Limitations

This study also has several limitations. First, each of the three studies relied on a single task with a single dependent variable to operationalize self-control. Although the present findings suggest that the moderating effect of sense of power on the relationship between disordered environments and self-control may arise from its influence on self-control resources, sense of control, and inhibitory control, these mechanisms were not directly tested. Future research could incorporate these variables simultaneously within a single study and employ event-related potentials (ERP) or functional magnetic resonance imaging (fMRI) to explicitly test the mediating mechanisms.

Furthermore, the participants in this study were exclusively university students, who typically have relatively limited experience with social power. Consequently, both the measured trait sense of power and the state sense of power manipulated via the recall task likely primarily reflected personal sense of power. Future studies could separately manipulate personal and social sense of power, or include participants from more diverse populations with varying power-related experiences.

Finally, the present research found that sense of power moderates the detrimental effect of disordered environments on self-control, pointing to a potential applied direction. Self-control is shaped by factors at multiple environmental levels (Hofmann, 2024). Sense of power, though measured here as an individual-level factor, is itself embedded within broader social structures (Anderson et al., 2012; Galinsky et al., 2003). This alignment raises the possibility of a multi-level intervention framework targeting sense of power. We emphasize, however, that this remains a speculative extension. The present studies tested only individual-level manipulations and did not examine multi-level interventions. Furthermore, all findings were obtained in controlled laboratory settings. We did not directly test the effectiveness of interventions aimed at boosting sense of power in real-world, ecologically complex settings, such as unhealthy eating or low physical activity. Future research could draw upon multi-level theoretical frameworks, such as the Four Levers of Policymaking Framework (4 LP; Hofmann et al., 2024), to design and test whether individual-level power manipulations are more effective when embedded within broader multi-level intervention frameworks.

## Conclusions

This study primarily yields the following conclusions:

1. Disordered environments impair individual self-control.
  Exposure to disordered environments leads individuals to prefer smaller immediate rewards, exhibit poorer behavioral persistence, and demonstrate reduced inhibitory control ability.
2. Sense of power moderates the detrimental effect of disordered environments on self-control.
  Trait sense of power provides a boundary condition for the effect: the impairing effect of disordered environments on self-control is confined to individuals with a low sense of power.
  State sense of power provides an intervention pathway: priming a high sense of power can protect individuals from the damaging effects of disordered environments on self-control.

## Data Availability

All study materials, datasets, and analysis code are available on the Open Science Framework (OSF) via this anonymous link: https://osf.io/dq7k9/?view_only=3ec3a52d8a47493c936974be23cb7dc5.
